# Polymorphism of gene cassette promoter variants of class 1 integron harbored in S. Choleraesuis and Typhimurium isolated from Taiwan

**DOI:** 10.7603/s40681-014-0020-3

**Published:** 2014-08-13

**Authors:** Chih-Sian Tseng, Yu-Chieh Yen, Chao-Chin Chang, Yuan-Man Hsu

**Affiliations:** 1 Department of Biological Science and Technology, College of Life Sciences, China Medical University, Taichung, Taiwan; 2 Graduate Institute of Microbiology and Public Health, School of Veterinary Medicine, National Chung Hsing University, Taichung, Taiwan; 3 China Medical University, 91, Hsueh-Shih Road, Taichung, Taiwan

**Keywords:** *Salmonella*, Class 1 integrons, Gene cassette, Promoter

## Abstract

Integrons, mobile genetic units, capture and incorporate antibiotic resistance gene cassette by site-specific recombination. Class 1 integrons are widespread and associated with dispersion of antibiotic resistance among Gram-negative bacteria. The expression of gene cassette in Class 1 can vary, based on the Pc promoter but seldom from another promoter hiding downstream of Pc, called P_2_. To probe distribution and prevalence of gene cassette promoter variants, we analyzed 169 *S*. Choleraesuis and 191 *S*. Typhimurium isolates from humans and animals, finding 95.27% occurrence of integrin among S. Choleraesuis, 83.25% among *S*. Typhimurium. PCR-RFLP analysis identified four promoters (PcS+P_2_, PcW_TGN-10_+P_2_, PcH1+P_2_, and Pc_WTGN-10_+P_2_-GGG) in said integron-positive isolates; major types in *S*. Choleraesuis and *S*. Typhimurium were PcS+P_2_ and Pc_WTGN-10_+P_2_, respectively. Likewise, β-galactosidase assay rated promoter strength of variants by transcriptional fusion constructs to show extended -10 promoter (TGn/-10 promoter) in Pc and three-nucleotide insertion (GGG) between -35 and -10 region of P_2_ improving promoter strength of gene cassette.

## 1. Introduction

Salmonellosis ranks among the most common bacterial infections worldwide [1]. Salmonella species are rod-shaped, aerobic, and Gram-negative bacteria, all major food-borne pathogens in the world [2]. Until 2004, over 2,500 Salmonella serotypes were identified [3]. Among these serovars, Salmonella enterica serovar Choleraesuis and Typhimurium are common non-typhoidal serotypes that pose global concern [4,5]. The USA diagnoses over 4 million cases of Salmonella infection per annum [6], about 500 fatal [2,7]. While mild and self-limited in adults, salmonellosis can require drugs, especially antibiotics, to treat infant, elderly, or immunocomprised patients [8]. Abuse of antibiotics in many locales nowadays spurs development of resistant strains. Studies show ever more multidrug resistance by Salmonella, causing serious public health hazards [5,9,10]. Such mechanisms entail obtaining genes or point mutation in genomes [11], resistance dispersed by [1] clonal expansion of drug-resistant strains or [2] horizontal transfer of determinants. Multidrug resistant genes transmitted between human and animal pathogens [12] mean mobile genetic elements playing a key role in dispersion of drug resistance among bacterial population [13-16].

Plasmids, transposons, and integrons are well known mobile genetic elements that mediate drug resistance genes disseminating via horizontal or vertical transfer [2]. Quantity of integron research has grown recently, with five classes of identified by sequences of integrases. Class 1 is most prevalent and closely linked with multidrug resistance in Gram-negative bacteria [11,17,18]. Typical Class 1 integron consists of intI gene encoding integrase, recombination specific site attI, major promoter Pc, and gene cassettes [18-21]. Over 100 gene cassettes harbored in Class 1 integron have been identified [22]; Pc is thought responsible for expression of gene cassettes [23]. Several Pc variants are described based on strength [24]: PcS for “strong”, PcW for “weak”, PcH1 for Hybrid 1, and PcH2 for Hybrid 2, the last two containing -35 and -10 hexamers of PcW and PcS in opposite combinations. Promoter strengths of PcH1 and PcH2 are intermediate between PcS and PcW. Studies indicate presence of “TGN” extended -10 motif between -35 and -10 hexamers raising transcription efficacy of σ70 promoters in E. coli [24,25]. Occasionally, Pc combines with a second promoter designated P_2_, located 119 bp downstream of Pc in 10% of Class 1 integrons [24,26,27]. A rare P_2_ type was described by Tenover [28] and Tae-Eun Kim [29]: three G residue insertion optimizes spacing (17 bp) between potential -35 and -10 hexamer sequences. Strength of four Pc types has been detailed in previous studies [24,30-32], several of which show a great polymorphism among variant Pc-P2 combinations. To evaluate dissemination of intergon-driven drug resistance, this study examined 360 Salmonella isolates for prevalence of Pc variants and strengths of Pc-P2 variant combinations in Taiwan.

## 2. Materials and methods

### 2.1. Bacterial strains and culture conditions

*Salmonella* isolates in this study had been described in previous report [5]. A total of 360 Salmonella isolates (169 S. Choleraesuis and 191 S. Typhimurium) were amassed from human and animal hosts. For serovar identification of Salmonella enterica, antiserum of O and H antigen detection were purchased from Denka Seiken Co., Ltd. in Japan and S&A Reagents Lab Limited in Thailand, respectively. Analysis based on the Kaufmann-White scheme and protocols for serotyping established by the Centers for Disease Control and Prevention in Atlanta, GA [33]. *Salmonella* isolates were maintained in 25 % frozen glycerol stock and inoculated on Salmonella-Shigella (SS) agar (Difco, USA) at 37°C.

### 2.2. PCR detection of Class 1 integrons in Salmonella isolates

All *Salmonella* isolates were probed for integrons by polymerase chain reaction (PCR) and nucleotide sequencing. After culturing bacteria in Luria-Bertani (LB) broth to log-stationary phase at 37 °C with vigorous shaking, genomic template DNA were extracted from isolates, as per manufacturer’s instructions for Tissue & Cell Genomic DNA Purification Kit (Genemark, Taiwan). Specific primers IntegronA and IntegronB [34] (Table 1) screened *intI1*, Class 1 integrase gene, within bacterial isolates. PCR mixture was in a total volume of 25 μl containing 3 μl genomic DNA as template, 1 μl of each primer (10 μM), 5 μl of 5x PCR Plus Master Mix II solution (Genemark, Taiwan), and 15 μl of distilled water. PCR mixture used T1 Thermocycler (Biometra, USA). Template was initially denatured at 95°C for 5 min followed by 35 cycles at 95°C for 30 sec, 57°C for 30 sec, and 72°C for 30 sec. Final extension for 10 min was done at 72 °C, PCR products confirmed via 1% agarose gel electrophoresis.

**Table 1:** 

Name^a^	Sequence (5’-3’)	Product size (bp)	References
IntegronA	GCCTTGCTGTTCTTCTACGG	558	[34]
IntegronB	GATGCCTGCTTGTTCTACGG		
SC-RGA-F1^b^	ATT**GGATCC**GGTGACGCACACCGTGGAAACGGAT	328	This study
SC-RGA-F2^b^	ATT**GGATCC**ACCTTGACCGAACGCAGCGGTGGTA	218	
SC-RGA-R^c^	ATTAAGCTTCGAGTTCATATGGCTAACTTTGTTT		

a. IntegronA and IntegronB primers detected Class I integron. SC-RGA-F1 and SC-RGA-R primers for Pc-P_2_ region in integrons (328 bp); SC-RGA-F2 and SC-RGA-R for only P_2_ region in integron (218 bp).

b. Nucleotide sequences recognized by *Bam*HI restriction enzyme underlined

c. Nucleotide sequences recognized by *Hind*III restriction enzyme underlined

### 2.3. Characterization of gene cassette promoter (Pc-P_2_) variants in Salmonella isolates by PCR-Restriction fragment length polymorphism (PCR-RFLP)

To ferret out promoter variants of Class 1 integrons in *Salmonella intI1*-positive isolates, PCR-RFLP method served for analysis: 330-bp fragment of Pc-P_2_ regions in Class 1 integrons amplified by PCR with SC-RGA-F1 and SC-RGA-R specific primers (Table 1). Preparation of PCR mixture as detailed above proceeded as follows: after initial denaturation (5 min at 95°C), DNA fragment was amplified for 35 cycles of 30 sec at 95°C, annealing at 60°C for 30 sec, and 30 sec for extension at 72°C, with a final extension step at 72°C for 7 min. Before performing RFLP, all PCR products were purified by PCR clean-up kit (Genemark, Taiwan). To screen promoter variants, HincII or AluI restriction enzymes identified Pc variants; BsrGI restriction enzyme was also applied to analyze three nucleotide insertions between -35 and -10 region of P_2_. Each digestion reaction containing 2 μl of 10X NEBuffer 4, 0.2 μl of 100X BSA, 1 μl of restriction enzyme (10U; New England BioLabs, Inc.), 10 μl of purified PCR products, and added distilled water to 20 μl. Mixture was incubated at 37°C for 6 hrs, after which treatments were analyzed on 1.5 % agarose gel electrophoresis.

### 2.4. Plasmid constructions for promoter activity assay

After characterizing types of gene cassette promoter, one bacterial strain stood for each type was picked randomly from Salmonella intI1-positive isolates. To study relative strength of a gene cassette promoter, transcriptional fusion with both Pc and P_2_ were cloned into the promoterless *lacZ* gene upstream in a reporter vector (pCB267, [35]). Extracting genomic DNA from bacterial strains representing promoter types, we amplified Pc-P_2_ region by specific SC-RGA-F1 and SC-RGA-R primers (Table 1). To gauge effect of three nucleotide insertions in -35 and -10 region of P_2_ on promoter strength, only P_2_ region with or without insertion was amplified by SC-RGA-F2 and SC-RGA-R primer pairs (Table 1). PCR were run for 5 min at 95°C followed by 35 cycles of 30 sec at 95°C, 30 sec at 60°C, 30 sec at 72°C and final extension of 15 min at 72°C. PCR products purified by kit (Genemark, Taiwan) were digested with *Bam*HI and *Hind*III, then ligated to *Bam*HI- and *Hind*III-digested pCB267. These constructs could assess strength of promoters.

### 2.5. β-galactosidase assay of promoter strength

Each recombinant plasmid carrying transcriptional fusion was transformed into *E. coli* strain DH5α to measure β-galactosidase enzyme activity, assays performed with 0.5-ml aliquots of overnight cultures as described [36, 37]. Bacteria cultured in LB broth containing 100 μg/ml of ampicillin at 37 °C were vigorously shaken overnight, collected by centrifuge, washed with Z buffer (60 mM Na_2_HPO_4_, 40 mM NaH_2_PO_4_, 10 mM KCl, and 50 mM β-mercaptoethanol, pH 7.0), and lysed by adding chloroform plus 0.1% SDS. Then 200 μl of 4 mg/ml ortho-nitrophenyl-β-galactoside (ONPG) substrate was added to reactants and incubated at 30°C, with incubation time recorded; 500 μl of 1M Na_2_CO_3_ served as stopper of the reaction. Value of OD420 was measured by spectrometer, units of β-galactosidase enzyme activity compared within constructs. Experiments were done at least three times per construct.

### 2.6. DNA sequencing

All PCR products represented different types of promoter and plasmids used for evaluating promoter strength were purified via PCR clean-up kit or plasmid miniprep purification kit (Genemark, Taiwan) and sequenced from both sides by AmpliTaq-FS DNA polymerase, dye terminator chemistry, and an automatic nucleic acid sequence analyzer (ABI Prism, USA) at the DNA sequence facility at Mission Biotech Co., Ltd. in Taiwan. Specific primers used for cloning and sequencing were synthesized by the same company. Nucleotide sequences were compared with the BLAST network in the GenBank database of National Center for Biotechnology Information (NCBI), U.S. National Library of Medicine [38].

### 2.7. Statistical analysis

All data of β-galactosidase enzyme activities were calculated at least three times, analyzed by commercial software SPSS Version 16 for Windows (SPSS Company; Chicago, IL). Pearson’s chi-square test derived linkage between independent groups, *p* value < 0.05 considered significant.

**Table 2:** 

**Promoter Type**	**Variant**	-35 Region		Spacing		-10 Region	
		Sequence	*HincII* (GTY/RAC)	No. of nt	N_14_-TCN (TGN)	Sequence	*AluI* (AG/CT)
P_C_	PcS	TTGACA	+	17	TCN	TAAACT	-
	PcW_TGN-10_	TGGACA	-	17	TGN	TAAGCT	+
	PcH1	TGGACA	-	17	TCN	TAAACT	-
**Promoter Type**	**Variant**	**-35 Region**	**Spacing**			**-10 Region**	
		Sequence	No. of nt	3-nt-insertion	*Bsr*GI (T/GTACA)	Sequence	
P_2_	P_2_	TTGTTA	14	-	+	TACAGT	
	P_2_-GGG	TTGTTA	17	+	-	TACAGT	

## 3. Results

### 3.1. Detection of Class 1 integrons among Salmonella isolates

All isolates in this study were characterized in previous work [5], including the Class 1 integron presence and antimicrobial resistance patterns. A total of 360 isolates, belonging to either S. Typhimurium or S. Choleraesuis, were collected from humans and animals during 1997-2009 in Taiwan. Among S. Choleraesuis isolates, 111 are from pigs and 50 from humans; among S. Typhimurium isolates, 17 are from pigs, 115 from humans, 1 from pigeons, 9 from turtles, 12 from chickens, 1 from snakes, 4 from ducks. Using PCR, we showed 95.27% (161/169) of S. Choleraesuis and 83.25% (159/191) of S. Typhimurium isolates harbor Class 1 integron (Table 2).

### 3.2. Molecular characterization of gene cassettes’ common promoters in intI1-positive Salmonella isolates

Pc is the major promoter located upstream of gene cassettes in Class 1 integron. Occasionally, a second promoter (P_2_) is located 119 bp downstream of Pc. Based on nucleotide sequence of -35 and -10 Pc regions, it can be classified into strong, hybrid, and weak types [24,27,29,32]. By driving the expression of CAT reporter with Pc variants, relative strengths of PcS and PcW + P_2_ to PcW were measured, with PcS and PcW+ P_2_ promoters about 30- and 15-fold higher than PcW, respectively [24,31].

PCR-RFLP characterized Pc-P_2_ variants. Promoter regions were amplified by primers SC-RGA-F1 and SC-RGA-R (Table 1), then amplicons subjected to enzyme digestion. The -35 region of PcS (TTGACA) and -10 region of PcW (TAAGCT) were digested by *Hinc*II and *Alu*I, respectively. Digestion of fragment containing -35 region of PcS with *Hinc*II would yield two fragments, 277bp and 51 bp in length; digestion of fragment containing -10 region of PcW with *AluI* would produce 252bp and 76 bp fragments. Those not fitting this restriction enzyme digestion pattern were subjected to sequence and classified as PcW_TGN-10_. Three guanine (GGG) insertions between -35 and -10 region of P_2_ were studied to create a new promoter (P_2_-GGG) occasionally found in Class 1 integrons [26]. For quicker P_2_ characterization, PCR amplified fragments were digested by *Bsr*G1. P2-GGG promoter could not be digested, but P_2_ promoter yielded fragments (188 and 140 bp) after digestion. Not only PCR-RFLP analysis but also DNA sequence was applied to variants, confirming promoters’ molecular characterization. Major Pc-P_2_ variants identified (Table 2) were PcS+P_2_, PcW_TGN-10_+ P_2_, PcH1+P_2_, and PcW_TGN-10_+P_2_-GGG.. PcS+P_2_ was the major variant in S. Choleraesuis (143/161, 88.82%) and PcW _TGN-10_+P_2_ for S. Typhimurium (131/159, 82.39%). Surprisingly, multiple variants appeared in PcS + P_2_/PcH1 + P_2_/PcW_TGN-10_ + P_2_ combination in S. Choleraesuis and PcW_TGN-10_ + P_2_/PcS + P_2_/PcW_TGN-10_ + P_2_-GGG combination in *S*. Typhimurium. Of six isolates, two of S. Choleraesuis and four of S. Typhimurium harbored more than one Pc-P*2* combination in one strain (Table 3).

**Table 3:** 

Serotype	Total no.	No. of integrons (%)	Promoter variant (occurrence, %)
*S*. Choleraesuis	169	161 (95.27 %)	PcS + P_2_ (143/161, 88.82 %) PcH1 + P_2_ (16/161, 9.94 %) PcS + P_2_/ PcH1 + P_2_/ PcW_TGN-10_ + P_2_ (2/161, 1.24 %)
*S*. Typhimurium	191	159 (83.25 %)	PcW_TGN-10_ + P_2_ (131/159, 82.39 %) PcS + P_2_ (15/159, 9.43 %) PcW_TGN-10_ + P_2_-GGG (9/159, 5.66 %) PcW_TGN-10_ + P_2_/ PcS + P_2_/ PcW_TGN-10_ + P_2_-GGG (4/159, 5.52 %)

**Figure 1:**
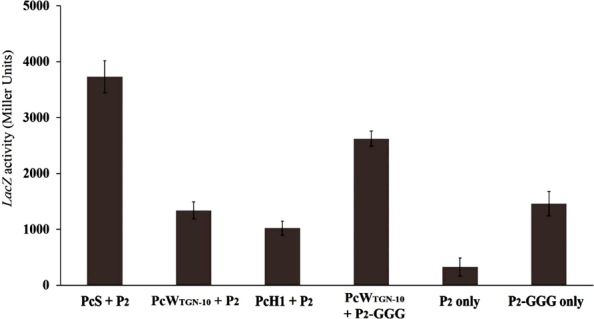


### 3.3. Relative strength of gene cassette promoter variants

To rate promoter strength, Pc-P_2_ each variant identified was cloned into a promoterless vector (pCB267) to drive *lacZ* reporter gene expression. β-galactosidase activities were compared between variants; PcS+P_2_ proved strongest (Fig. 1). Strength of PcW_TGN-10_ + P_2_ was 31% greater than PcH1+P_2_. In prior studies, strength of hybrid Pc had intermediate activity between PcS and PcW [24]. Promoter activity of PcW rises with PcW carrying TGN-10 motif between -35 and -10 region (PcW_TGN-10_). Strength of P_2_-GGG region alone was 4.5-fold that of P_2_ without insertion. Our data concurred with earlier studies [28, 29]: promoter strength of PcW_TGN-10_ combined with P_2_-GGG was about 2-fold that of PcW_TGN-10_ combined with P_2_. Therefore, 3-G insertion can enhance promoter strength in general.

## 4. Discussion

*S*. Typhimurium DT104, a bacterial strain isolated during the early 1980s in the United Kingdom, is resistant to multiple antibiotics: e.g., ampicillin, chloramphenicol, streptomycin, sulfonamides, tetracycline (ACSSuT). After that, multi-drug resistance to ACSSuT is a common *Salmonella* trait [5, 39-41]. ACSSuT resistance genes are mostly disseminated through Class 1 integron [5]. In our study, 88.9% of *Salmonella* isolates harbored Class 1 integron in Taiwan. Comparing two serovars, positive rate of Class I integron in S. Choleraesuis (95.27 %) was higher than in S. Typhimurium (83.25 %)., suggesting Class 1 integron already widespread in Taiwan.

PCR-RFLP analysis identified four Pc-P_2_ combinations: PcS+P_2_, PcW_TGN-10_+P_2_, PcH1+P_2_, and PcW_TGN-10_+P_2_-GGG., the first two predominant in *S*. Choleraesuis and *S*. Typhimurium, respectively. These variants in our study are also the most prevalent forms in silico study [24]. Based on previous research on gene cassettes in Class 1 integron [5], for isolates carrying more than one set of Pc-P_2_ combination, some only carry one kind of gene cassette. Data portend more than one copy of Class 1 integrons in one strain, albeit with variant promoter combination.

Transcriptional fusion constructs were used to monitor promoter strength among Pc-P_2_ variants. Our data agreed with prior study that strengths of promoter variants is PcS > PcW_TGN-10_ > PcH1. Besides, our data showed TGN-10 motif between -35 and -10 region of Pc and three nucleotide insertions (GGG) between -35 and -10 region of P_2_ boosting both promoter strength and expression of gene cassettes [24]. Sequencing analysis avers that most isolates carrying PcH1+ P_2_ variant have guanine located 11bp downstream of -10 region of P_2_, but adenine could also occur. Still, no significant difference appeared between strengths of PcH1 and mutated PcH1 variants, showing single mutation of this site as random and not affecting expression of gene cassettes. In sum, integrons are well-known machinery to spread bacterial genetic elements, especially antibiotic resistance genes. This research proved Pc highly polymorphic; its strength may affect downstream gene cassette expression. Promoter polymorphism might alter levels of bacterial antibiotic resistance in response to environmental stress.

## Acknowledgments

This study was funded by Grants CMU102-S-11 from China Medical University and 102-2313-B-005-008-MY2 from the National Science Council, Taiwan.

**Declaration of Interest:** Authors declare no conflicts of interest for this work.
